# A Protocol for Reconstructing Complex Lateral Skull Base Defects: Two Decades of Tertiary Care Experience

**DOI:** 10.1002/ohn.70321

**Published:** 2026-06-23

**Authors:** Muhammad Umar Farooq, Yuri Hirayama, Jameel Muzaffar, Raghu Kumar, Charlie Huins, Peter Monksfield, James Baden, Demetrius Evriviades, Richard Irving

**Affiliations:** ^1^ Department of Ear, Nose and Throat Surgery–Otology & Skull Base Surgery University Hospitals Birmingham NHS Foundation Trust Birmingham UK; ^2^ Department of Applied Health Sciences University of Birmingham Birmingham UK; ^3^ Madras ENT Research Foundation Chennai India; ^4^ Department of Plastic & Reconstructive Surgery University Hospitals Birmingham NHS Foundation Trust Birmingham UK

**Keywords:** otology, reconstruction, temporal bone

## Abstract

**Objective:**

To describe a multidisciplinary protocol for the resection and reconstruction of complex lateral skull base pathology, based on over two decades of experience in a high‐volume tertiary referral center in the United Kingdom.

**Study Design:**

Retrospective descriptive analysis of institutional practice.

**Setting:**

A high‐volume tertiary skull base unit in the United Kingdom.

**Methods:**

This study presents a summary of clinical experience in managing lateral skull base pathologies—both benign and malignant—over a 20‐year period. It outlines a multidisciplinary approach involving ENT, neurosurgery, plastic surgery, anesthesia, radiology, oncology, and specialist nursing teams. The institutional protocol for resection and reconstruction is described, including surgical planning, intraoperative decision‐making, perioperative care, and long‐term rehabilitation strategies.

**Results:**

The integrated multidisciplinary protocol enabled safe and effective management of a broad range of lateral skull base lesions. The team achieved high rates of complete resection and functional reconstruction, with acceptable levels of morbidity. Complex anatomy, tumor extent, and reconstructive requirements were successfully navigated through coordinated team‐based planning. The approach has been refined through iterative experience and now serves as a reference pathway for similarly structured centers.

**Conclusions:**

Effective management of lateral skull base pathology requires a highly coordinated multidisciplinary strategy. The described protocol, developed and implemented over two decades, offers a structured and reproducible approach that may serve as a valuable reference for other high‐volume centers involved in skull base surgery.

The lateral skull base, encompassing intricate neurovascular structures and serving as the barrier between cranial and extracranial regions, poses significant surgical and reconstructive challenges when disrupted by pathological processes or by the removal of anatomical barriers during surgical access to the pathology. Surgical removal of pathologies involving the temporal bone, whether benign or malignant, can lead to extensive defects, necessitating meticulous reconstruction to restore functional integrity. These pathologies span a wide spectrum, from locally invasive benign processes such as cholesteatomas and paragangliomas to aggressive malignancies, including squamous cell carcinomas (SCCs), each presenting distinct challenges in terms of resection and reconstruction. The aggressive nature of some of these pathologies, particularly SCC, necessitates radiotherapy, potentially resulting in osteoradionecrosis (ORN). Management of ORN itself may warrant excision and reconstruction in severe cases, with the principal aim being to restore blood flow to the diseased facial nerve. While skull base surgeons typically focus on complete resection of disease, reconstruction of the defect requires adequate coverage of vital structures whilst restoring structure and function.^1^

Advances in surgical techniques and multidisciplinary team (MDT) management have improved patient outcomes; however, the reconstructive management of composite lateral temporal bone defects remains complex and challenging to skull base teams. This is primarily due to the surgical excision involving the wide resection of bony tissues, skin, auricle, parotid, infratemporal fossa as well as extension into dura leaving behind exposed cranial nerves, major vessels, and brain; all of which can complicate reconstruction due to the anatomical intricacy, the need to prevent cerebrospinal fluid (CSF) leaks, and the restoration of both cosmetic and functional aspects, including hearing and facial nerve integrity.^2^

Effective reconstruction of lateral temporal bone defects necessitates the use of vascularised, robust, and predictable tissue in sufficient quantities to minimize significant complications, including CSF leaks, meningitis, intracranial sepsis, stroke, seizures, lower cranial nerve deficits affecting airway, voice and swallowing, and potential mortality.^3^ In Rosenthal et al, critical reconstructive goals for addressing lateral temporal bone defects were outlined, namely: protecting cervical vascular structures, restoring appropriate facial contour, and preventing CSF leaks. They proposed a classification system to streamline reconstructive strategies into three distinct categories:

- Class I: defects involve superficial structures, such as the pre‐auricular skin, portions of the auricle, or the parotid gland.
- Class II: defects include preservation of part of the auricle with limited cutaneous involvement.
- Class III: defects represent more extensive resections, including complete auricular removal (pinnectomy) alongside lateral temporal bone resection.^4^

Muscle and musculocutaneous flaps have emerged as optimal choices for reconstructing these defects due to their ability to provide substantial tissue volume, facilitating reliable and watertight closure. Historically, pedicled regional flaps were frequently employed; however, concerns regarding their reliability and the challenges posed by patient positioning have led to the widespread adoption of free flaps as the preferred reconstructive modality. This evolution reflects advancements in reconstructive strategies aimed at improving functional and esthetic outcomes while mitigating the risks associated with complex skull base defects.^5^

Despite these advancements, consensus on a standardized approach to managing such defects is limited, and outcomes often vary based on institutional expertise, surgical preferences, and patient‐specific factors. Due to the heterogeneity of the pathology encountered, there is limited evidence in literature to formulate a standard protocol for lateral skull base reconstruction.^6^

This study describes the long‐term experience of a tertiary referral center in the United Kingdom, focusing on the reconstruction of lateral skull base defects arising from diverse pathologies. By analyzing outcomes from a substantial and varied patient cohort, this study seeks to identify key principles and propose a practical, evidence‐based, multi‐disciplinary protocol for achieving safe and reliable results in managing temporal bone defects. It is hoped that these findings will inform clinical decision‐making, optimize reconstructive strategies, and ultimately improve quality of life for patients facing challenging clinical presentations.

## Methods

A retrospective review of patient charts was conducted for individuals who underwent primary resection and reconstruction of the temporal bone for a wide variety of lateral skull base pathologies at a UK tertiary center from February 2004 to 2023. This, involving human participants, adhered to the ethical standards set by the institutional and national research committee, as well as the 1964 Helsinki Declaration, including its subsequent amendments or equivalent ethical guidelines. Approval for this study was granted by the University Hospitals Birmingham NHS Foundation Trust Quality Improvement Department (Clinical Audits and Registries Management Services (CARMS) registration number: 21664). The study did not require formal ethics committee review after completing the National Decision Tool developed by the Medical Research Council Regulatory Support Centre in partnership with the Health Research Authority.^7^ The data was accessed between May 1, 2024 and June 1, 2024 and no identifiable patient data were used.

Patients were identified from the electronic patient record system based on predefined inclusion and exclusion criteria. All patients who were referred to the regional skull base MDT with pathology of the temporal bone and subsequently underwent temporal bone resection followed by any form of reconstruction for benign or malignant pathologies were included in the study. All patients underwent resection and reconstruction on the same day with multidisciplinary input from Otolaryngologist and Plastic and Reconstructive Surgeons. Conversely, cases were excluded if they represented duplicate entries, or if patient data were incomplete or missing critical details related to either the surgical procedure or follow‐up outcomes.

Demographic and clinical data were recorded, including patient age, sex, histological diagnosis, cancer stage, and surgical indications. Tumors were staged according to their specific cancer type, following the classification guidelines of the American Joint Committee on Cancer (AJCC). The analysis included the extent of lateral temporal bone resection, the status of neck dissection, the type of reconstruction performed, and details of adjuvant therapies such as chemotherapy, radiotherapy, or chemoradiation. Postoperative outcomes were evaluated, focusing on the occurrence of surgical complications, disease‐free survival, and overall survival. Additional parameters assessed included the duration of follow‐up and the incidence of specific complications, such as wound infection, ORN, and wound healing issues.

## Results

In this study, a heterogenous cohort of patients is presented, owing to the differences in pathology and time of presentation. A total of 115 patients (77 males, 38 females) between 2004 and 2024 with varying later skull base pathologies were included. Patient ages ranged from 18 to 91 years, with a mean age of 63.4 years. Individual patient level data for the total cohort have been presented in Supplementary Material 1, available online.

Of the total cohort, 57.4% (n = 66) underwent extensive lateral temporal bone excision for malignancy. The remaining patients (n = 49) were treated for benign conditions. SCC was the most common diagnosis, accounting for 45.2% of cases (n = 53). Advanced petrous cholesteatomas represented 13% (n = 15), while other carcinomas (eg, adenocarcinoma, basal cell carcinoma, and carcinoma ex pleomorphic adenoma) constituted 12.2% (n = 14). Schwannomas (vestibular and trigeminal) accounted for 5.2% (n = 6). The remaining 27 cases (23.5%) included a variety of rare and non‐cancerous conditions.

For cases involving primary malignancies, benign pathology, or ORN, surgical resections varied in complexity based on the underlying pathology and disease extent. All surgical plans were meticulously developed during local skull base MDT meetings to ensure tailored and precise interventions. Petrousectomy, temporal bone resection, or pinnectomy represented 32% (n = 37) of excisional approaches. Parotidectomy or mastoid exploration was performed in 12% of cases (n = 14). The most extensive resections, comprising total pinnectomy, lateral temporal bone resection, and neck dissection, were carried out in 56% of patients (n = 64) (Table 1). The choice of surgical approach was guided by the aggressiveness of the disease and the need for complete oncological clearance or symptom management.

**Table 1:** Extent of Lateral Temporal Bone Excision

Excision/Defect	No. of patients
Petrousectomy/Temporal bone resection/Pinnectomy	37 (32%)
Parotidectomy/Mastoid exploration	14 (12%)
Total Pinnectomy/Lateral Temporal Bone Resection/Neck Dissection +	64 (56%)

**Figure 1:**
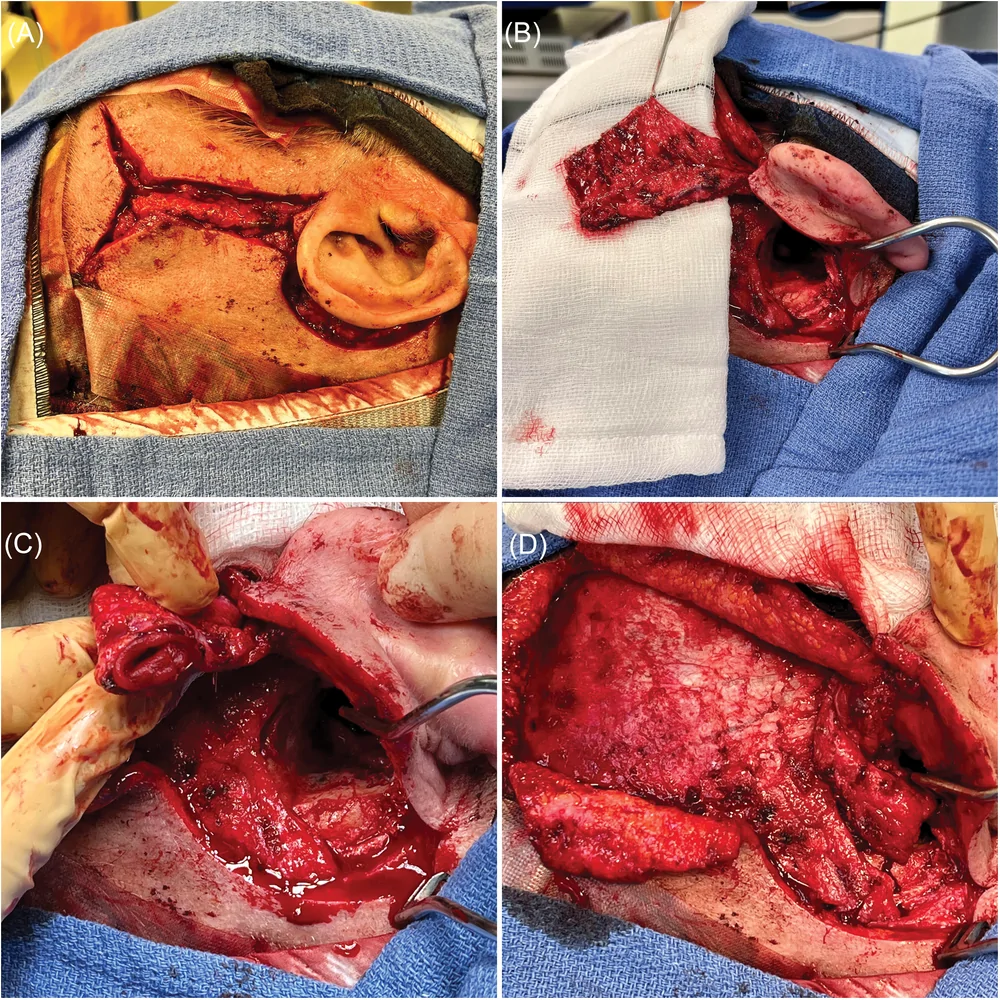
Large skull base defect post‐pinnectomy prior to 3D reconstruction. (A) Access incision for temporoparietal fascia (TPF) flap; (B) TPF flap elevated for Integra; (C) TPF flap augmented, rolled Swiss‐roll; (D) Transposed into mastoid defect.

Reconstruction using vascularised flaps was achieved in 85.2% (n = 98) of patients, while 14.8% (n = 17) did not require flap‐based reconstruction. In the case of 2 patients with malignancy of the parotid gland, vascularised flaps were not deemed appropriate due to the presenting pathology. These patients required facial nerve reconstruction only in contrast to bone, nerve or dura coverage. Among those who received vascularised reconstructions, the anterolateral thigh (ALT) flap was the most commonly employed technique, used in 38.8% (n = 38) of patients. This was followed by the temporoparietal fascia (TPF) flap in 24.5% (n = 24). Abdominal fat grafts were the reconstruction choice for 16.3% (n = 16) patients. Radial forearm, gracilis, serratus anterior, pectoralis major, or latissimus dorsi (LD) flaps were used in 10.4% (n = 12). Abdominal fat grafts were utilized in 16 patients (16.3%), and the pectoralis major flap was used in 2 patients (2.0%). Other techniques—such as free fibular flap—accounted for the remaining eight cases (8.2%). The choice of reconstruction method was dictated by the defect's complexity, location, and the patient's clinical requirements (Figure 1).

Facial nerve palsy (House‐Brackmann grade 2‐6) was encountered in 21.7% (n = 25) of the cohort. The facial nerve was sacrificed in 34 cases where complete facial palsy was seen clinically due to disease infiltration. Where possible the nerve was primarily repaired with a direct ipsilateral nerve graft connecting the facial stump to the facial branches which was performed in 12 (10.4%) patients like sural nerve or greater auricular nerve grafts, and with nerve transfers in 2 (1.7%) patients, such as masseteric nerve grafts. In addition, other facial reanimation therapy like eyelid gold weights, botulinum toxin, and fascia lata slings were implemented in 14 cases with good effect.

Post‐operative outcomes highlighted the challenges associated with temporal bone resection and reconstruction. Post‐operative complications were documented in several cases. Flap failure occurred in 7% (n = 8), requiring additional interventions in some instances. Wound haematomas were identified in 7.8% (n = 9), while infections were noted in 5.2% (n = 6) cases. A CSF leak, though uncommon, was reported in a single case (0.9%), necessitating careful monitoring and management.

Where appropriate, adjuvant therapy was used to manage malignancies. Radiotherapy was the most common representing 33.9% (n = 39), followed by chemoradiation at 7.8% (n = 9) and stereotactic radiosurgery at 1.7% (n = 2). Only 3.5% (n = 4) of the cohort presented with ORN with an average of 37.0 months to reach ORN.

Cancer recurrence was observed amongst 22.6% (n = 26) of the cohort, with 17.4% (n = 20) presenting as local recurrence and 5.2% (n = 6) as distant metastases. Of the 40.9% (n = 47) of patients who succumbed during the study period, the average time to mortality post‐surgery was approximately 13.9 months (patient level data for all mortalities has been presented in Supplementary Material 2, available online). Despite these outcomes, the mean follow‐up duration for the cohort was substantial at 63.9 months, underscoring the long‐term commitment to monitoring and care. This experience has helped to infer that there is a limited window of opportunity for the skull base team to rationally approach the pathology for appropriate resection and reconstruction. If there is disease recurrence despite the best of efforts, further options remain limited and the morbidity/mortality rates become more significant.

## Discussion

This retrospective study highlights the complexity and challenges associated with temporal bone resection and reconstruction in a heterogeneous cohort of 115 patients with diverse skull base pathologies. This experience has relied on practical contact and observation of these patients from Plastic Surgery, Otolaryngology, and Oral and Maxillofacial Surgery. The findings demonstrate the need for precise surgical planning, multidisciplinary collaboration, and tailored reconstruction techniques to achieve optimal outcomes.

Temporal bone resection was largely required for patients predominantly presenting with malignancies. SCC of the temporal bone accounts for less than 0.2% of cancers in the head and neck region; however, it is implicated as the most common neoplasm of the external auditory canal.^8^ What is established in literature also aligns with the findings of this study where SCC was the most common malignancy (45.2%), which underscores SCC as the primary pathology in temporal bone malignancies due to its aggressive nature and predilection for local invasion.^9^ The high incidence of malignancy in this cohort (57.4%) reflects the importance of oncological clearance, which necessitates complex surgical approaches including total pinnectomy, lateral temporal bone resection, and neck dissection performed in over half of the patients (56%).

The role of vascularised flap reconstruction was critical in this cohort, with 85.2% undergoing such procedures. The ALT flap emerged as the most commonly used reconstructive option (38.8%), followed by TPF flaps (24.5%) and abdominal fat grafts (16.3%). This distribution is consistent with the current consensus favoring ALT flaps for their versatility, robust vascular supply, and ability to accommodate large defects.^10^, ^11^ The ALT flap, first described by Song et al in 1984, has become a favored option for reconstruction due to its reliability and versatility. It offers a large skin area and can be harvested as either a fascio‐cutaneous or a musculocutaneous flap, with thickness and volume easily customized to fit the defect.^12^ However, the choice of flap was influenced by defect size, anatomical location, and patient‐specific factors, as seen in the use of less common options like gracilis, serratus, and pectoralis major flaps.^13^

Despite the success of reconstructive techniques, post‐operative complications were significant. Flap failure occurred in 7% of patients, a rate comparable to Wong et al's case series.^14^ Similarly, haematomas and infections, noted in 7.8% and 5.2% of cases respectively, highlight the importance of vigilant post‐operative monitoring and early intervention. A single case of CSF leak underscores the risks associated with temporal bone resection, particularly in proximity to critical structures like the dura.

Reconstruction following lateral skull base resection demands meticulous planning to address the complexity of the defect, considering factors such as volume restoration and functional requirements. This study underscores the critical need for a standardized protocol in temporal bone resection and reconstruction, incorporating comprehensive preoperative planning, multidisciplinary input, and individualized surgical and reconstructive approaches. Over many years of expertise at our institution, we have developed a structured algorithm (Table 2) to evaluate defect size and extent, ensuring optimal flap selection based on tissue volume needed for coverage, functional restoration, and esthetic outcomes.

**Table 2:** The Birmingham Protocol for 3D Reconstruction of Lateral Skull Base Defects Due to Osteoradionecrosis (ORN) and Temporal Bone Malignancy

3D reconstruction for cancer	Hierarchy of management for osteoradionecrosis
*Small bone defects with no skin loss* Temporo‐parietal fascial flap (TMPFF) +/− Integra	*Stage 1 (limited ORN)*: Regular Aural Toilet with Antibiotics cover (topical/oral/IV) +/− Hyperbaric O_2_ therapy (10 × 100% O_2_ for 90 min at 2.4 ATA)
*Moderate volume defects with no skin loss* Gracilius or Serratus Anterior or Latissimus Dorsi Muscle Flap	*Stage 2 (Refractory ORN)*: Local debridement—Canalplasty, Meatoplasty, Mastoidectomy +/− Hyperbaric O_2_ therapy (10 × 100% O_2_ for 90 min at 2.4 ATA)
*Skin loss with moderate/large defect* Anterolateral Thigh with Vastus Lateralis chimeric flap	*Stage 3 (FULMINANT ORN)* Petrosectomy + Blind sac closure with abdominal fat graft (ENT) TMJ/Mandibular Procedures (Maxillofacial surgeons) Lateral Temporal Bone Resection + Dural Repair (ENT/Neurosurgeons) Vascularized Tissue Transfers: Local Pedicled Flaps/Free Flaps (Plastics)

For small bone defects restricted to the tympano‐mastoid region without skin loss, the superficial TPF flap was employed, enhanced with one to three layers of Integra to augment volume and reposition the pinna. This approach was effective in 24 patients (24.5%) with no petrosectomy required.

Moderate defects extending to the middle fossa, posterior fossa, skull base foramina, or styloid process were reconstructed using gracilis, serratus, or LD free flaps, as these did not require additional skin.

**Figure 2:**
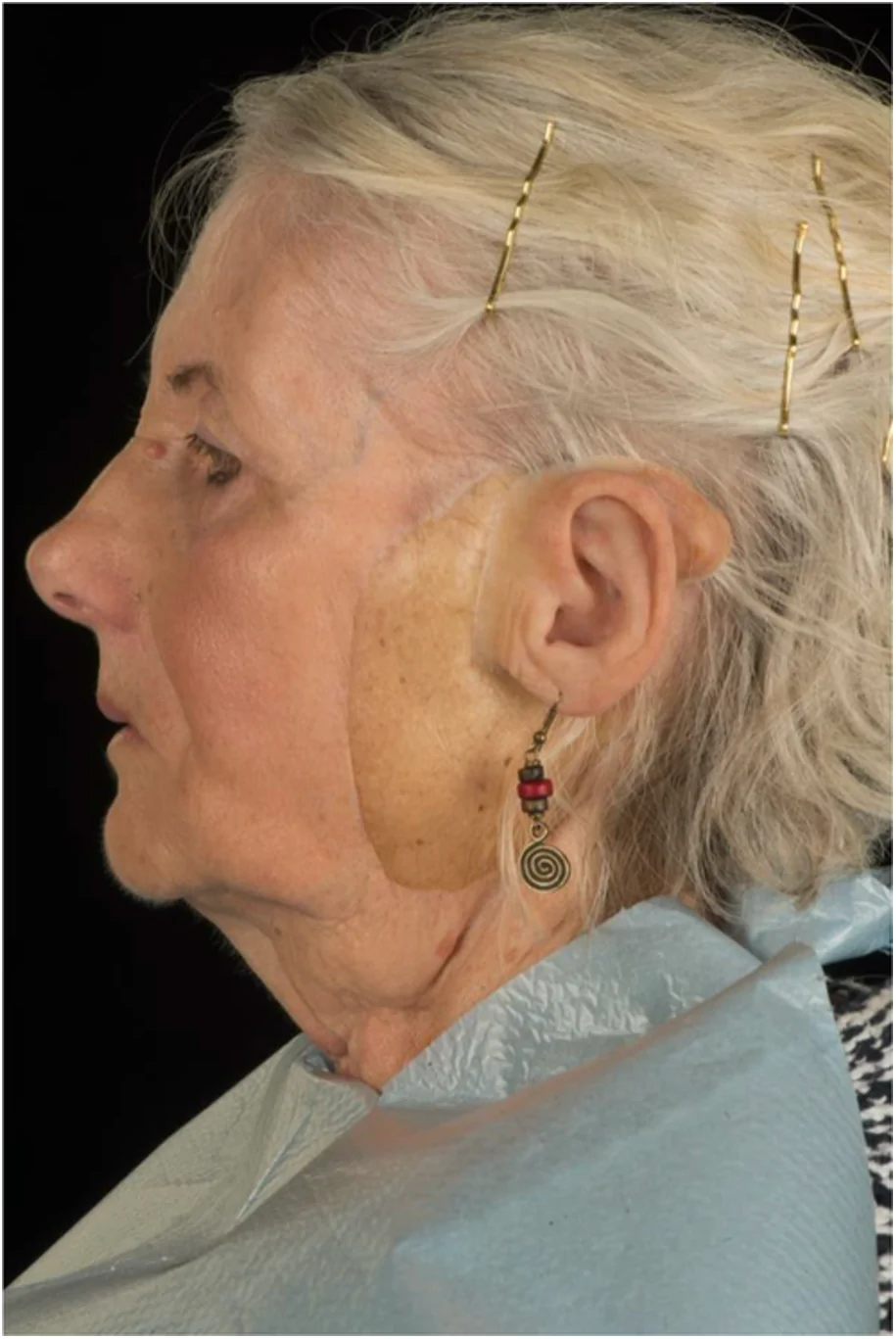
After reconstruction with anterolateral thigh (VL chimeric flap) patient was also fitted with bone anchored artificial prosthetic ear (Vistafix; Cochlear LTD) and bone anchored hearing aid (Ponto; Oticon Medical). VL, lastus lateralis.

For extensive defects involving the entire temporal bone, adjacent structures, and at times intracranial tissues, the ALT flap was utilized in 38 cases (33.8%). These cases often included total pinnectomy and temporal bone resection with exposed dura and the risk of CSF leaks. In patients requiring total pinnectomy or middle ear sacrifice, hearing restoration was achieved intraoperatively using bone conduction implants and osseointegrated fixtures for custom prosthetic ears (Figure 2).

Management of temporal bone ORN remains a challenge due to its potential for severe complications, including neuropathies and intracranial sepsis. The Birmingham protocol (Table 2) follows a structured approach to managing ORN, emphasizing early conservative measures such as hyperbaric oxygen therapy and antibiotic coverage, while reserving radical surgical intervention for refractory cases. This aligns with findings by Richards et al, who advocate for a multidisciplinary strategy incorporating radical skull base surgery and vascularised tissue transfer for advanced ORN cases. Their retrospective review further reinforces the necessity of standardized protocols to optimize outcomes in patients with extensive disease requiring lateral temporal bone resection.^15^

**Figure 3:**
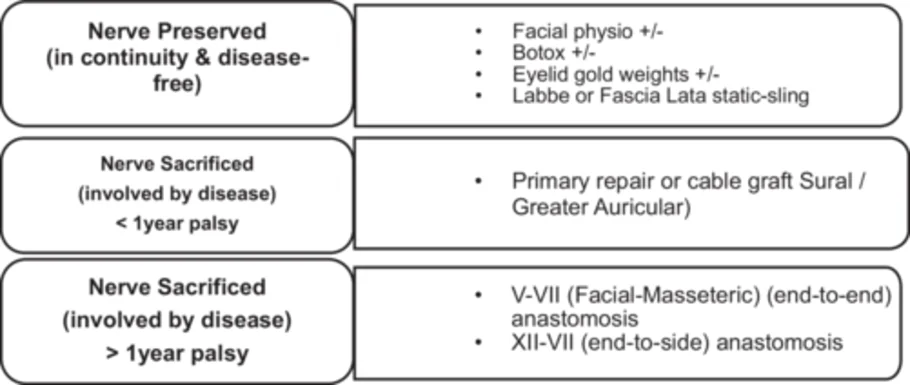
Facial reanimation principle.

The hierarchy of facial nerve repair was adopted in cases where it was imperative the nerve sacrificed or when a complete facial palsy was evident pre‐operatively presumably due to disease infiltration (Table 3). Facial nerve repair was prioritized when necessary, with strategies including direct grafts, nerve transfers, and adjunctive reanimation techniques such as eyelid weights and fascia lata slings (Figure 3).

**Table 3:** Hierarchy of Facial Nerve Repair

Hierarchy of facial nerve repair
Primary repair if possible
Primary repair after re‐routing
Grafting with total bed preparation
Static sling/Labbe dependent on patient factors
Masseteric nerve transfer in preference to graft if > 1 year palsy

Postoperative care involved speech and language therapy (SALT), facial physiotherapy, and tailored rehabilitation, integral components of our multidisciplinary management protocol. Long‐term surveillance continues to assess outcomes in hearing restoration, facial cosmesis, and motor function, ensuring early identification and management of complications. The proposed protocol is now embedded in institutional guidelines and consistently applied by the skull base MDT. Future directions include expanding prospective data collection for multicentre studies, enabling larger cohort analyses to provide higher‐quality evidence. Collaborative efforts among high‐volume centers can support a nationwide audit of outcomes, potentially driving uniform practice changes across the United Kingdom.

## Conclusion

Reconstructing complex lateral skull base defects needs multidisciplinary expertise in order to achieve optimal structural and functional outcomes, with minimal morbidity. Each aspect of reconstruction, including preserving vital structures, facial reanimation, hearing, and cosmetic prosthesis fixation needs meticulous planning and execution and can be provided by tertiary care hospitals with dedicated skull base units.

Two decades of experience in managing lateral skull base defects with a systematic multidisciplinary approach in a high‐volume tertiary center in the United Kingdom has helped propose a useful protocol. Such an algorithm should provide safe, consistent, and successful disease‐free reconstruction with minimal morbidity, despite the diverse heterogeneity of the primary pathology encountered.

## Author Contributions

**Muhammad Umar Farooq,** methodology, investigation, data curation, formal analysis, writing (original draft), writing (review and edit); **Yuri Hirayama,** methodology, investigation, data curation, formal analysis, writing (review and edit); **Jameel Muzaffar,** conceptualization, methodology, formal analysis, writing (review and edit), visualization, project administration, supervision; **Raghu Kumar,** conceptualization, methodology, formal analysis, writing (review and edit), visualization, project administration; **Charlie Huins,** methodology, formal analysis, writing (review and edit); **Peter Monksfield,** methodology, formal analysis, writing (review and edit); **James Baden,** methodology, formal analysis, writing (review and edit); **Demetrius Evriviades,** methodology, formal analysis, writing (review and edit); **Richard Irving,** methodology, formal analysis, writing (review and edit).

## Disclosures

### Competing interests

None.

### Funding source

None.

## Supporting information

Supplementary Material 1: Individual patient level data for all participants.

Supplementary Material 2: Individual patient level data for all mortalities of the cohort.
