# Supplementary material for: A Protocol for Reconstructing Complex Lateral Skull Base Defects: Two Decades of Tertiary Care Experience
Source: Otolaryngol Head Neck Surg. 2026 Jun 23;175(3):587–93. doi: 10.1002/ohn.70321 (PMC13525878; doi:10.1002/ohn.70321)
Supplement: Supplementary file 1 [file OHN-175-587-s001.docx]

| **No.** | **Age** | **Sex** | **Significant comorbidity** | **Pathology** | **Primary Site** | **Adjuvant Therapy** | **Complications** | **Mortality** | **Months to mortality** | **Cause of mortality** |
| --- | --- | --- | --- | --- | --- | --- | --- | --- | --- | --- |
| 1 | 47 | F | Hypothyroidism | Squamous cell carcinoma | Pinna | None | None | Deceased | 19 | Not documented |
| 2 | 82 | M | Stroke, hypertension | Squamous cell carcinoma | Pinna | None | None | Deceased | 127 | Not documented |
| 3 | 70 | F | Not documented | Cholesteatoma | Data not available | None | None | Deceased | 12 | Not documented |
| 4 | 57 | F | Von Hippel Landau syndrome | Endolymphatic sac tumour | Posterior semi-circular canal | None | None | Deceased | 52 | Intracranial bleed |
| 5 | 71 | F | Stroke | Vestibular schwannoma | None | None | None | Deceased | 96 | Ischaemic bowel |
| 6 | 48 | M | Asthma | Parotid adenoid cystic carcinoma | Parotid | None | None | Deceased | 51 | Disease recurrence |
| 7 | 64 | F | Von Willeband Disease IIa | Squamous cell carcinoma | External auditory canal | Radiotherapy | None | Deceased | 163 | Spontaneous haemorrhage |
| 8 | 65 | M | None | Ex-pleomorphic adenoma and acinic carcinoma | Parotid | Radiotherapy | None | Deceased | 39 | Not documented |
| 9 | 62 | F | Not documented | Basal cell carcinoma | Pinna | None | None | Deceased | 83 | Peritoneal metastatic disease |
| 10 | 76 | M | Heart failure, Parkinson’s disease | Squamous cell carcinoma | Conchal bowl | None | None | Deceased | 0 | Pneumonia |
| 11 | 84 | F | Waldenstrom’s macrogloulinaemia, Crohn’s disease, non-Hodgkin’s lymphoma | Squamous cell carcinoma | External auditory canal | Radiotherapy | None | Deceased | 5 | Not documented |
| 12 | 88 | F | Hypertension, vascular dementia | Basal cell carcinoma | External auditory canal | None | None | Deceased | 25 | Not documented |
| 13 | 80 | M | Alzheimer’s disease | Squamous cell carcinoma | External auditory canal | Radiotherapy | None | Deceased | 23 | Not documented |
| 14 | 75 | F | Hypertension | Basal cell carcinoma | Helix | None | None | Deceased | 96 | Not documented |
| 15 | 73 | M | Hypertension, TIA, pulmonary embolism | Squamous cell carcinoma | Parotid | Radiotherapy | None | Deceased | 66 | Not documented |
| 16 | 59 | M | Previous amputation | Squamous cell carcinoma | External auditory canal | Radiotherapy | None | Deceased | 19 | Not documented |
| 17 | 62 | M | Alcoholic liver disease, T2DM | Squamous cell carcinoma | Lower lip | Chemoradiation | None | Deceased | 10 | Disease recurrence |
| 18 | 84 | M | Hypertension | Squamous cell carcinoma | Pinna | None | None | Deceased | 27 | Not documented |
| 19 | 80 | M | IHD, hypertension | Adenocarcinoma | Parotid | None | Wound breakdown | Deceased | 9 | Disease recurrence |
| 20 | 80 | M | COPD, hypertension | Carcinoma ex pleomorphic adenoma | Parotid | None | None | Deceased | 50 | Not documented |
| 21 | 73 | M | Hypertension, hypercholesterolaemia, T2DM, Crohn’s disease | Squamous cell carcinoma | Post-auricular | Radiotherapy | None | Deceased | 5 | Brain metastatic disease |
| 22 | 59 | M | Kaposi’s sarcoma | Osteosarcoma | Post-auricular area | None | Wound haematoma | Deceased | 13 | Brain metastatic disease |
| 23 | 76 | M | Hypertension, T2DM | Squamous cell carcinoma | External auditory canal | Radiotherapy | None | Deceased | 13 | Disease recurrence |
| 24 | 66 | F | Asthma | Angiosarcoma (metastasis) | Ear | Radiotherapy | None | Deceased | 24 | Disease recurrence |
| 25 | 66 | M | Atrial fibrillation | Ductal carcinoma | Parotid | Radiotherapy | None | Deceased | 21 | Brain metastatic disease |
| 26 | 86 | M | Hypertension | Squamous cell carcinoma | Pinna | Radiotherapy | None | Deceased | 71 | Not documented |
| 27 | 81 | M | Hypertension, T2DM | Squamous cell carcinoma | Pinna | None | None | Deceased | 10 | Not documented |
| 28 | 79 | M | T2DM | Squamous cell carcinoma (metastasis) | Forehead | Radiotherapy | Skin necrosis of the flap + wound infection | Deceased | 3 | Pulmonary metastatic disease |
| 29 | 77 | M | Hypertension | Squamous cell carcinoma | External auditory canal | None | None | Deceased | 23 | Not documented |
| 30 | 80 | M | Hypertension, glaucoma | Squamous cell carcinoma | Pinna | None | None | Deceased | 29 | Renal metastatic disease |
| 31 | 84 | M | None | Carcinoma | Parotid | Radiotherapy | None | Deceased | 28 | Pulmonary metastatic disease |
| 32 | 56 | M | Hypertension | Squamous cell carcinoma | External auditory canal | Chemoradiation | None | Deceased | 32 | Brain metastatic disease |
| 33 | 78 | M | Hypertension | Squamous cell carcinoma | External auditory canal | None | None | Deceased | 14 | Not documented |
| 34 | 57 | M | None | Malignant melanoma | Temporal bone | Chemoradiation | None | Deceased | 4 | Pulmonary metastatic disease |
| 35 | 87 | M | None | Squamous cell carcinoma | Pinna | None | None | Deceased | 1 | Pneumonia |
| 36 | 76 | M | Hypothyroidism | Adenocarcinoma | Parotid | Radiotherapy | Wound haematoma | Deceased | 7 | Pelvis metastatic disease |
| 37 | 83 | F | Rheumatoid arthritis | Squamous cell carcinoma | External auditory canal | None | None | Deceased | 2 | Not documented |
| 38 | 66 | M | Hypertension, hypercholesterolaemia | Carcinoma ex pleomorphic adenoma | Parotid | Radiotherapy | None | Deceased | 13 | Not documented |
| 39 | 86 | F | Hypertension | Basal cell carcinoma | External auditory canal | Radiotherapy | None | Deceased | 24 | Not documented |
| 40 | 56 | M | Hypertension, T2DM | Squamous cell carcinoma | External auditory canal | Chemoradiation | None | Deceased | 4 | Pulmonary metastatic disease |
| 41 | 86 | M | T2DM, IHD, atrial fibrillation, CKD 3 | Squamous cell carcinoma | Pinna | None | Post-operative haematoma | Deceased | 8 | Not documented |
| 42 | 73 | M | COPD, hypercholesterolaemia | Squamous cell carcinoma | External auditory canal | None | None | Deceased | 4 | Disease recurrence |
| 43 | 83 | M | None | Squamous cell carcinoma | Pinna | None | None | Deceased | 127 | Not documented |
| 44 | 53 | F | Not documented | Squamous cell carcinoma | External auditory canal | Radiotherapy | None | Deceased | Not documented | Not documented |
| 45 | 77 | M | Hypertension | Squamous cell carcinoma | External auditory canal | None | None | Deceased | 8 | Not documented |
| 46 | 84 | F | Waldenstrom’s macroglobulinaemia, Crohn’s disease, non-Hodgkin’s lymphoma | Squamous cell carcinoma | Skull base | None | None | Deceased | 17 | Not documented |
| 47 | 45 | M | None | Carcinoma | Tonsil | Chemoradiation | None | Deceased | 20 | Disease recurrence |
