# Supplementary material for: A Protocol for Reconstructing Complex Lateral Skull Base Defects: Two Decades of Tertiary Care Experience
Source: Otolaryngol Head Neck Surg. 2026 Jun 23;175(3):587–93. doi: 10.1002/ohn.70321 (PMC13525878; doi:10.1002/ohn.70321)
Supplement: Supplementary file 2 [file OHN-175-587-s002.docx]

| **No.** | **Age** | **Sex** | **Significant comorbidity** | **Pathology** | **Primary Site** | **Adjuvant Therapy** | **Complications** | **Mortality** | **Months to mortality** | **Cause of mortality** |
| --- | --- | --- | --- | --- | --- | --- | --- | --- | --- | --- |
| 1 | 71 | M | Atrial fibrillation, heart failure, hypertension | Cholesteatoma | Petrous apex | None | None | Alive | NA | NA |
| 2 | 47 | F | Hypothyroidism | Squamous cell carcinoma | Pinna | None | None | Deceased | 19 | Not documented |
| 3 | 35 | F | Hypertension | Cholesteatoma | Petrous apex | None | None | Alive | NA | NA |
| 4 | 70 | M | Not documented | Cholesteatoma | Middle ear | None | None | Alive | NA | NA |
| 5 | 56 | M | Atrial fibrillation, hypertension, chronic lymphoid leukaemia | Squamous cell carcinoma | Pinna | Radiotherapy | None | Alive | NA | NA |
| 6 | 82 | M | Stroke, hypertension | Squamous cell carcinoma | Pinna | None | None | Deceased | 127 | Not documented |
| 7 | 40 | M | None | Trigeminal schwannoma | Petrous apex | Stereotactic radiosurgery | None | Alive | NA | NA |
| 8 | 70 | F | Not documented | Cholesteatoma | Data not available | None | None | Deceased | 12 | Not documented |
| 9 | 24 | M | None | Endolymphatic sac tumour | Internal auditory canal | None | None | Alive | NA | NA |
| 10 | 66 | M | Atrial fibrillation, hypertension | Vestibular schwannoma | Internal auditory meatus | None | Otitis externa, granulation polyp | Alive | NA | NA |
| 11 | 49 | M | Myeloma | Cholesteatoma | Petrous apex | None | Abdominal wall haematoma (GA evacuation). Post auricular discharge | Alive | NA | NA |
| 12 | 44 | M | Asthma, epilepsy | Vestibular schwannoma | Internal auditory meatus | Stereotactic radiosurgery | Otalgia/neuralgia | Alive | NA | NA |
| 13 | 57 | F | Von Hippel Landau syndrome | Endolymphatic sac tumour | Posterior semi-circular canal | None | None | Deceased | 52 | Intracranial bleed |
| 14 | 28 | M | None | Cholesteatoma | Geniculate ganglion | None | None | Alive | NA | NA |
| 15 | 29 | F | Iron deficiency anaemia | Cholesteatoma | Middle ear | None | None | Alive | NA | NA |
| 16 | 49 | M | Hypertension | Cholesteatoma | Petrous apex | None | None | Alive | NA | NA |
| 17 | 18 | M | None | Recurrent meningitis - pneumatisation of internal ear | None | None | None | Alive | NA | NA |
| 18 | 55 | F | Hypertension, PVD, T2DM, COPD, osteoporosis | Vestibular schwannoma | None | None | Skin necrosis | Alive | NA | NA |
| 19 | 71 | F | Stroke | Vestibular schwannoma | None | None | None | Deceased | 96 | Ischaemic bowel |
| 20 | 76 | F | Asthma | Cholesteatoma | Petrous apex | None | None | Alive | NA | NA |
| 21 | 53 | F | None | Granuloma post failed stapedectomy (otosclerosis) | Middle ear | None | None | Alive | NA | NA |
| 22 | 71 | F | None | Cholesteatoma | Petrous apex | None | CSF leak | Alive | NA | NA |
| 23 | 62 | M | None | Cholesteatoma | Facial ridge | None | Infected abdominal fat graft, sigmoid sinus thrombosis, sepsis within cerebellum, cerebellum oedema | Alive | NA | NA |
| 24 | 48 | F | Rheumatoid arthritis | Cholesterol granuloma | Petrous apex | None | Recurrence of cyst | Alive | NA | NA |
| 25 | 73 | F | Glaucoma, spina bifida | Cholesteatoma | Petrous apex | None | Wound dehiscence (minimal) | Alive | NA | NA |
| 26 | 35 | M | None | Mucosal disease | Middle ear | Radiotherapy | None | Alive | NA | NA |
| 27 | 50 | F | Hypertension, hypercholesterolaemia | Mucoepidermoid carcinoma | Middle ear | Radiotherapy | None | Alive | NA | NA |
| 28 | 48 | M | Asthma | Parotid adenoid cystic carcinoma | Parotid | None | None | Deceased | 51 | Disease recurrence |
| 29 | 55 | M | Hypertension, Crohn’s disease | Cholesteatoma | Petrous apex | None | None | Alive | NA | NA |
| 30 | 22 | F | None | Cholesteatoma | Petrous apex | None | None | Alive | NA | NA |
| 31 | 32 | M | None | Vestibular schwannoma | Internal auditory meatus | Radiotherapy | None | Alive | NA | NA |
| 32 | 56 | M | Glaucoma | Cholesteatoma | Retrofacial | None | None | Alive | NA | NA |
| 33 | 28 | M | T2DM | Vestibular schwannoma | Internal auditory meatus | Radiotherapy | Vocal cord paralysis | Alive | NA | NA |
| 34 | 33 | M | Hypertension, hypercholesterolaemia | Chondrosarcoma | Extradural | Proton beam therapy | Wound infection | Alive | NA | NA |
| 35 | 58 | F | Trigeminal neuralgia | Chondrosarcoma | Temporo-mandibular joint | Radiotherapy | None | Alive | NA | NA |
| 36 | 64 | F | Von Willeband Disease IIa | Squamous cell carcinoma | External auditory canal | Radiotherapy | None | Deceased | 163 | Spontaneous haemorrhage |
| 37 | 65 | M | None | Ex-pleomorphic adenoma and acinic carcinoma | Parotid | Radiotherapy | None | Deceased | 39 | Not documented |
| 38 | 48 | M | TIA, atrial flutter, IHD, T2DM | Squamous cell carcinoma | External auditory canal | Radiotherapy | None | Alive | NA | NA |
| 39 | 62 | F | Not documented | Basal cell carcinoma | Pinna | None | None | Deceased | 83 | Peritoneal metastatic disease |
| 40 | 76 | M | Heart failure, Parkinson’s disease | Squamous cell carcinoma | Conchal bowl | None | None | Deceased | 0 | Pneumonia |
| 41 | 85 | M | Atrial fibrillation, heart failure, PVD, T2DM | Basal cell carcinoma | Pinna | None | None | Alive | NA | NA |
| 42 | 84 | F | Waldenstrom’s macrogloulinaemia, Crohn’s disease, non-Hodgkin’s lymphoma | Squamous cell carcinoma | External auditory canal | Radiotherapy | None | Deceased | 5 | Not documented |
| 43 | 88 | F | Hypertension, vascular dementia | Basal cell carcinoma | External auditory canal | None | None | Deceased | 25 | Not documented |
| 44 | 66 | M | Not documented | Squamous cell carcinoma | External auditory canal | None | None | Alive | NA | NA |
| 45 | 80 | M | Alzheimer’s disease | Squamous cell carcinoma | External auditory canal | Radiotherapy | None | Deceased | 23 | Not documented |
| 46 | 75 | F | Hypertension | Basal cell carcinoma | Helix | None | None | Deceased | 96 | Not documented |
| 47 | 78 | M | T2DM, CKD, COPD, hypercholesterolaemia | Squamous cell carcinoma | External auditory canal | Immunotherapy | Flap failure and necrosis | Alive | 26 | NA |
| 48 | 71 | M | Heart failure, atrial fibrillation | Squamous cell carcinoma | Pinna | Radiotherapy | Infection donor ALT site | Alive | NA | NA |
| 49 | 91 | M | Hypertension, IHD, T2DM, hypercholesterolaemia | Squamous cell carcinoma | Pinna | None | None | Alive | 78 | NA |
| 50 | 70 | M | Hypertension, non-diabetic hyperglycaemia | Adenocarcinoma | External auditory canal | Radiotherapy | None | Alive | NA | NA |
| 51 | 70 | F | Hypertension, T2DM, hypothyroidism, hypercholesterolaemia | Adenoid cystic carcinoma | External auditory canal | Radiotherapy | Wound haematoma | Alive | NA | NA |
| 52 | 43 | F | Pre-diabetes, diverticular disease | Adenocarcinoma | External auditory canal | None | None | Alive | NA | NA |
| 53 | 73 | M | Hypertension, TIA, pulmonary embolism | Squamous cell carcinoma | Parotid | Radiotherapy | None | Deceased | 66 | Not documented |
| 54 | 66 | M | T2DM | Squamous cell carcinoma | External auditory canal | Radiotherapy | None | Alive | NA | NA |
| 55 | 83 | F | Not documented | Squamous cell carcinoma | External auditory canal | None | Wound haematoma | Alive | NA | NA |
| 56 | 40 | F | None | Adenoid cystic carcinoma | Parotid | Radiotherapy | Wound breakdown, granulating tissue | Alive | NA | NA |
| 57 | 68 | F | None | Squamous cell carcinoma | External auditory canal | Chemoradiation | Wound abscess | Alive | NA | NA |
| 58 | 56 | M | Hypertension, tuberculosis | Squamous cell carcinoma | External auditory canal | None | Division of venous pedicle | Alive | NA | NA |
| 59 | 49 | F | Hypertension, hypercholesterolaemia | Squamous cell carcinoma | External auditory canal | Chemoradiation | Skin necrosis of the flap + discharging wound | Alive | NA | NA |
| 60 | 59 | M | Previous amputation | Squamous cell carcinoma | External auditory canal | Radiotherapy | None | Deceased | 19 | Not documented |
| 61 | 24 | M | None | Adenocarcinoma | Paranasal sinuses | Radiotherapy | Right petrosectomy defect | Alive | NA | NA |
| 62 | 62 | M | Alcoholic liver disease, T2DM | Squamous cell carcinoma | Lower lip | Chemoradiation | None | Deceased | 10 | Disease recurrence |
| 63 | 84 | M | Hypertension | Squamous cell carcinoma | Pinna | None | None | Deceased | 27 | Not documented |
| 64 | 70 | M | Hypertension, end-stage renal disease | Squamous cell carcinoma | Pinna | Radiotherapy | Failed osseintegrated screws | Alive | NA | NA |
| 65 | 78 | M | Hypertension, T2DM, benign prostatic hyperplasia | Sebaceous carcinoma | External auditory canal | None | None | Alive | NA | NA |
| 66 | 50 | F | Hypertension, cerebral venous sinus thrombosis | Squamous cell carcinoma | External auditory canal | Chemoradiation | Failed osseointegrated implant | Alive | NA | NA |
| 67 | 80 | M | IHD, hypertension | Adenocarcinoma | Parotid | None | Wound breakdown | Deceased | 9 | Disease recurrence |
| 68 | 80 | M | COPD, hypertension | Carcinoma ex pleomorphic adenoma | Parotid | None | None | Deceased | 50 | Not documented |
| 69 | 73 | M | Hypertension, hypercholesterolaemia, T2DM, Crohn’s disease | Squamous cell carcinoma | Post-auricular | Radiotherapy | None | Deceased | 5 | Brain metastatic disease |
| 70 | 59 | M | Kaposi’s sarcoma | Osteosarcoma | Post-auricular area | None | Wound haematoma | Deceased | 13 | Brain metastatic disease |
| 71 | 76 | M | Hypertension, T2DM | Squamous cell carcinoma | External auditory canal | Radiotherapy | None | Deceased | 13 | Disease recurrence |
| 72 | 66 | F | Asthma | Angiosarcoma (metastasis) | Ear | Radiotherapy | None | Deceased | 24 | Disease recurrence |
| 73 | 47 | F | None | Leiomyosarcoma | Parotid | Radiotherapy | None | Alive | NA | NA |
| 74 | 66 | M | Atrial fibrillation | Ductal carcinoma | Parotid | Radiotherapy | None | Deceased | 21 | Brain metastatic disease |
| 75 | 86 | M | Hypertension | Squamous cell carcinoma | Pinna | Radiotherapy | None | Deceased | 71 | Not documented |
| 76 | 81 | M | Hypertension, T2DM | Squamous cell carcinoma | Pinna | None | None | Deceased | 10 | Not documented |
| 77 | 79 | M | T2DM | Squamous cell carcinoma (metastasis) | Forehead | Radiotherapy | Skin necrosis of the flap + wound infection | Deceased | 3 | Pulmonary metastatic disease |
| 78 | 77 | M | Hypertension | Squamous cell carcinoma | External auditory canal | None | None | Deceased | 23 | Not documented |
| 79 | 71 | M | Benign prostatic hypertrophy, hypothyroidism | Squamous cell carcinoma | External auditory canal | Radiotherapy | None | Alive | NA | NA |
| 80 | 80 | M | Hypertension, glaucoma | Squamous cell carcinoma | Pinna | None | None | Deceased | 29 | Renal metastatic disease |
| 81 | 84 | M | None | Carcinoma | Parotid | Radiotherapy | None | Deceased | 28 | Pulmonary metastatic disease |
| 82 | 56 | M | Hypertension | Squamous cell carcinoma | External auditory canal | Chemoradiation | None | Deceased | 32 | Brain metastatic disease |
| 83 | 78 | M | Hypertension | Squamous cell carcinoma | External auditory canal | None | None | Deceased | 14 | Not documented |
| 84 | 57 | M | None | Malignant melanoma | Temporal bone | Chemoradiation | None | Deceased | 4 | Pulmonary metastatic disease |
| 85 | 87 | M | None | Squamous cell carcinoma | Pinna | None | None | Deceased | 1 | Pneumonia |
| 86 | 76 | M | Hypothyroidism | Adenocarcinoma | Parotid | Radiotherapy | Wound haematoma | Deceased | 7 | Pelvis metastatic disease |
| 87 | 76 | F | Hypertension, CKD | Carcinoma ex-ploemorphic adenoma | Parotid | Radiotherapy | None | Alive | NA | NA |
| 88 | 83 | F | Rheumatoid arthritis | Squamous cell carcinoma | External auditory canal | None | None | Deceased | 2 | Not documented |
| 89 | 91 | F | Hypertension, epilepsy, IBS | Melanoma | Pinna | None | None | Alive | NA | NA |
| 90 | 66 | M | Hypertension, hypercholesterolaemia | Carcinoma ex pleomorphic adenoma | Parotid | Radiotherapy | None | Deceased | 13 | Not documented |
| 91 | 86 | F | Hypertension | Basal cell carcinoma | External auditory canal | Radiotherapy | None | Deceased | 24 | Not documented |
| 92 | 67 | M | Hypercholesterolaemia | Acinic cell carcinoma | Parotid | Radiotherapy | None | Alive | NA | NA |
| 93 | 41 | M | None | Adenocarcinoma | Parotid | Radiotherapy | None | Alive | NA | NA |
| 94 | 73 | M | Hypertension | Squamous cell carcinoma | Post-auricular | Radiotherapy | None | Alive | NA | NA |
| 95 | 61 | F | None | Squamous cell carcinoma | External auditory canal | None | Collection at left axilla (donor site) | Alive | NA | NA |
| 96 | 77 | M | Hypertension | Squamous cell carcinoma | Parotid | Radiotherapy | Skin breakdown and exposed necrotic bone | Alive | NA | NA |
| 97 | 61 | M | None | Basal cell carcinoma | Pinna | None | None | Alive | NA | NA |
| 98 | 45 | F | Pre-diabetes | Adenocarcinoma | External auditory canal | Radiotherapy | Infection | Alive | NA | NA |
| 99 | 56 | M | Hypertension, T2DM | Squamous cell carcinoma | External auditory canal | Chemoradiation | None | Deceased | 4 | Pulmonary metastatic disease |
| 100 | 59 | M | Hypertension, pre-diabetes | Carcinoma in situ Squamous cell carcinoma | External auditory canal | None | None | Alive | NA | NA |
| 101 | 64 | M | Glaucoma, prostate cancer | Squamous cell carcinoma | External auditory canal | Chemoradiation | Wound swelling and leaking | Alive | NA | NA |
| 102 | 86 | M | T2DM, IHD, atrial fibrillation, CKD 3 | Squamous cell carcinoma | Pinna | None | Post-operative haematoma | Deceased | 8 | Not documented |
| 103 | 78 | M | T2DM, CKD, COPD, hypercholesterolaemia | Squamous cell carcinoma | External auditory canal | Immunothrapy | None | Alive | NA | NA |
| 104 | 73 | M | COPD, hypercholesterolaemia | Squamous cell carcinoma | External auditory canal | None | None | Deceased | 4 | Disease recurrence |
| 105 | 82 | M | T2DM | Basal cell carcinoma | Pinna | Radiotherapy | None | Alive | NA | NA |
| 106 | 88 | F | End-stage renal disease | Squamous cell carcinoma | External auditory canal | None | None | Alive | NA | NA |
| 107 | 55 | M | Hypertension, hypercholesterolaemia | Squamous cell carcinoma | External auditory canal | None | None | Alive | NA | NA |
| 108 | 57 | M | Hypertension, epilepsy, non-alcoholic fatty liver disease | Squamous cell carcinoma | External auditory canal | Radiotherapy | Failure of flap | Alive | NA | NA |
| 109 | 56 | M | Hypertension, hypercholesterolaemia, atrial fibrillation | Squamous cell carcinoma | Pinna | Radiotherapy | None | Alive | NA | NA |
| 110 | 83 | M | None | Squamous cell carcinoma | Pinna | None | None | Deceased | 127 | Not documented |
| 111 | 53 | F | Not documented | Squamous cell carcinoma | External auditory canal | Radiotherapy | None | Deceased | Not documented | Not documented |
| 112 | 66 | M | None | Squamous cell carcinoma | Pinna | None | Wound haematoma | Alive | NA | NA |
| 113 | 77 | M | Hypertension | Squamous cell carcinoma | External auditory canal | None | None | Deceased | 8 | Not documented |
| 114 | 84 | F | Waldenstrom’s macroglobulinaemia, Crohn’s disease, non-Hodgkin’s lymphoma | Squamous cell carcinoma | Skull base | None | None | Deceased | 17 | Not documented |
| 115 | 45 | M | None | Carcinoma | Tonsil | Chemoradiation | None | Deceased | 20 | Disease recurrence |
